# Prevalence and Impact of Substance Use on Hospitalization and Post-Discharge Outcomes in Individuals with Congestive Heart Failure: Findings from a Safety-Net Hospital †

**DOI:** 10.3390/ijerph22121832

**Published:** 2025-12-07

**Authors:** Rosemarie Majdalani, Asmaa AlShammari, Marie Thearle, Mariel Magdits, Jinal Shah, Natalia Ionescu, Damian Kurian, Farbod Raiszadeh

**Affiliations:** 1 California Health Sciences University College of Osteopathic Medicine, Clovis, CA 93611, USA; 2 The Institute of Human Nutrition, Columbia University Irving Medical Center, New York, NY 10032, USA; 3 Harlem Hospital Center, Health + Hospitals, Vagelos College of Physicians and Surgeons, Columbia University, New York, NY 10037, USA; 4 Dasman Diabetes Institute, P.O Box 1180, Dasman 15462, Kuwait; 5 Colorado School of Public Health, Aurora, CO 80045, USA

**Keywords:** substance use, congestive heart failure, socioeconomic status

## Abstract

**Introduction**: Congestive heart failure (CHF) is a major global health challenge and the leading cause of hospitalization in the U.S., with disproportionately high 30-day readmission rates among low-income and minority communities. Social drivers of health and substance use both influence CHF outcomes, yet the effect of substance use on short-term readmissions remains understudied. This study evaluated the association between substance use and all-cause 30-day readmissions at a Safety-Net Community Hospital. **Methods**: A retrospective chart review was conducted among 500 adults admitted with CHF between 2019 and 2021. Substance use was defined as any documented use, identified through a positive urine toxicology or patient-reported social history of cocaine, tetrahydrocannabinol (THC), opioids, amphetamines, benzodiazepines, barbiturates, methadone, or phencyclidine (PCP). Alcohol and tobacco were assessed separately. Group differences were assessed using Chi-square and Wilcoxon rank-sum tests. Multivariable logistic regression was used for 30-day readmissions, and multivariable Poisson regression was used for total hospitalizations and length of stay (LOS). **Results**: Evidence of substance use was present in 38% of patients, with cocaine and THC most common. Patients with a history of substance use were younger, more often male, and experienced greater socioeconomic disadvantage. They had higher all-cause 30-day readmissions (21% vs. 14%; *p* = 0.048), more total hospitalizations (median 2 vs. 1 stay; *p* < 0.0001), and shorter LOS (median 4 vs. 5 days; *p* = 0.04). No differences were observed in 7-day post-hospitalization. **Conclusions**: Substance use is common among CHF patients at a safety-net hospital and was associated with higher 30-day readmissions as well as shorter hospital stays, which may reflect premature discharge rather than improved recovery. Future studies should assess whether addressing substance use alongside socioeconomic disparities can reduce readmissions in this population.

## 1. Introduction

Congestive heart failure (CHF) affects an estimated 5.7 million people in the United States and 64.3 million people worldwide, making it a leading cause of hospitalization and readmission [1,2,3,4]. In the United States, CHF admissions rose from 1.27 million in 1979 to 3.86 million in 2004, and nearly 80 percent of people hospitalized for CHF are readmitted within five years [1,3]. Annual costs reached $30.7 billion in 2012 and are projected to exceed $69.8 billion by 2030 [4].

Social determinants of health (SDoH)—the conditions in which people are born, live, learn, work, and age—critically influence CHF outcomes [5]. Social drivers of health such as lower socioeconomic status, structural racism, and inequitable access to healthcare and nutritious foods contribute to both the development and progression of CHF [2,6,7,8]. These inequities contribute to higher rates of readmission and mortality in historically underserved communities, particularly among African American and Hispanic adults, who are disproportionately affected by CHF-related morbidity and mortality [6,7,8,9]. These inequities manifest in multiple ways. Poverty may limit access to medications, transportation, and stable housing, while food insecurity and neighborhood disadvantage can restrict adherence to dietary recommendations and self-management strategies for CHF. Such factors not only worsen outcomes directly but also increase vulnerability to substance use, further compounding risk in populations already carrying a disproportionate CHF burden.

Substance use represents an additional and often intersecting risk factor. Alcohol, tobacco, opioids, and stimulants can exacerbate cardiac dysfunction and are associated with higher rates of hospitalization among patients with CHF [10,11,12,13,14,15,16]. Yet, the impact of substance use on short and long-term readmissions remains poorly characterized, particularly in safety-net hospitals where the overlap of SDoH and substance is most pronounced.

This study aimed to examine the prevalence of substance use among CHF patients and its association with 30-day and total hospital readmissions, length of stay, and follow-up appointment attendance, accounting for demographic and socioeconomic factors. Characterizing these patterns in a safety-net hospital population can inform future research and interventions to improve outcomes among historically underserved adults with CHF.

## 2. Methods

### 2.1. Study Design

This retrospective chart review was approved by the Biomedical Research Alliance of New York (BRANY) Institutional Review Board (IRB) and conducted at Harlem Hospital Center as part of an ongoing initiative to characterize CHF outcomes in a real-world, safety-net hospital population. The study included all patients (N = 500) admitted with a primary diagnosis of CHF or CHF-related complications between 1 January 2019, and 31 December 2021. The index admission was defined as the first hospitalization during the study period with a qualifying diagnosis, which served as the reference point for 30-day readmissions, total hospitalizations, and length of stay. For patients with multiple admissions, only the first qualifying hospitalization was considered the index admission.

### 2.2. Study Population

Patients were identified through multiple sources: the hospital’s EHR (EPIC), the Care Management Department’s database, and the American Heart Association’s Get With The Guidelines–Heart Failure (GWTG-HF) monthly billing list. Diagnosis confirmation was achieved through review of cardiology consultations, medical team notes, laboratory tests, and echocardiograms. No patients meeting these criteria were excluded. The sample size reflects all patients with confirmed CHF admitted during the study period. Analyses were planned at a predetermined sample size of 500, which provided >80% power to detect a medium effect (w = 0.3) for comparisons with a group of at least 88.

### 2.3. Data Collection and Variables

Data collected included demographics, clinical comorbidities, urine toxicology results, alcohol and tobacco use, socioeconomic variables (income, government assistance, family support, and housing), length of stay (LOS), hospital readmissions, and cardiology clinic attendance. Evidence of substance use was defined as either a positive urine toxicology (UTOX) or patient-reported social history for barbiturates, benzodiazepines, cocaine, methadone, opiates, phencyclidine (PCP), tetrahydrocannabinol (THC), or amphetamines. Toxicology testing and social history assessments were performed as part of routine clinical care. Alcohol consumption and cigarette smoking were evaluated separately from other substances, relying exclusively on the social history section of the EHR. 

Variables were measured according to their appropriate scale: substance use, 30-day readmission, sex, and race/ethnicity were nominal; socioeconomic indicators were categorical; and age, total hospitalizations, and LOS (days) were ratio variables. All clinical and utilization outcomes were extracted from the EHR and the institutional CHF Registry, which undergo routine quality checks for accuracy and completeness and are widely considered reliable measures. Covariates such as comorbidities were identified via ICD-10 codes and chart review, while demographic and socioeconomic data were derived from patient-reported admission history, which provides important context despite potential reporting bias. Substance use measures combined standardized toxicology testing with social history documentation, ensuring strong face validity.

### 2.4. Outcomes

The study had two aims: (a) to describe the prevalence of substance use among individuals hospitalized with CHF, and (b) to examine the association of substance use with hospitalization and follow-up outcomes. Outcomes for our second aim included 30-day all-cause readmission, total readmissions, and length of stay (LOS). Attendance at cardiology outpatient appointments within 7 days of discharge was extracted from the EHR. This variable was evaluated descriptively to assess attrition differences between individuals with and without a history of substance use and was included as a covariate in the multivariable Poisson regression model predicting total readmissions to account for potential differences in post-discharge care. 

### 2.5. Statistical Analysis

Continuous variables were described as mean (standard deviation) for normally distributed variables and median [interquartile range] for skewed variables. Categorical variables were described as frequencies (percentages). Statistical differences between groups were assessed using Chi-squared tests or Fisher’s exact tests for categorical variables or Wilcoxon rank-sum tests for continuous variables.

Covariates included in the analyses were selected based on clinical relevance and prior literature linking these factors to CHF outcomes and hospital readmissions [1,2,3,4]. Age, sex, race/ethnicity, BMI, and chronic kidney disease were included in the multivariable models for 30-day readmissions because they are established risk factors for CHF complications [4].

A multivariable logistic regression model was used to assess the impact of substance use on 30-day readmission rates after accounting for age, sex, race, BMI, and chronic kidney disease. Multivariable Poisson regression models were used to assess the relationship of substance use with LOS and total readmissions using the same covariates with the addition of whether a cardiology clinic visit occurred for the model exploring total readmissions. Statistical analyses were conducted in R (version 4.4.2) using two-sided *p*-values with an alpha of 0.05. Given the study’s exploratory design, no corrections were made for multiple comparisons.

Given the time period of data collection, sensitivity analyses were performed to account for COVID-19. Models were re-estimated after adjusting for a history of COVID-19 infection or vaccination prior to or concurrent with the index hospitalization, as well as after excluding the three individuals with a 30-day readmission due to COVID-19. To further assess the robustness of the findings, additional analyses incorporated smoking status, given its strong correlation with substance use in our data and its established contribution to cardiovascular disease risk. Although not prespecified, these analyses were undertaken to strengthen confidence in the observed associations, particularly for LOS.

## 3. Results

### 3.1. Baseline Demographics

Baseline demographic characteristics of the 500 CHF patients are summarized in Table 1. On average, the study population was older (age: 65 ± 13) with 56% males and 76% who identified as African American. A large majority (90%) had comorbid hypertension and a significant proportion had obesity (48%), diabetes mellitus (46%), dyslipidemia (46%), or renal disease (43%). CHF was diagnosed during the index hospitalization for 36% of the study population.

### 3.2. Prevalence of Substance Use

Evidence for substance use was prevalent in 38% of patients (189 individuals), of whom 134 (70.9%) individuals had evidence by positive urine toxicology and 124 (65.6%) individuals had evidence by self-report during the social history (69 (36.5%) patients met both criteria). Details of the prevalence for individual substances as determined by both urine toxicology and social history reports in patients with CHF are in Table 2. Cocaine and THC had the highest prevalence (62% and 63%, respectively). Patients were most likely to report cocaine, THC, or opiate use.

### 3.3. Patient Characteristics by Substance Use

Differences between those who did or did not have a history of substance use are shown in Table 1 and Table 3. Patients with a history of substance use were, on average, younger (mean age 60 ± 10 vs. 69 ± 14 years; *p* < 0.001), more likely to be male (66% vs. 50%; *p* = 0.0003), and had a lower BMI (30 vs. 32 kg/m^2^; *p* = 0.04) compared to those with no evidence of substance use. Patients with evidence of substance use were more likely to rely on Medicaid (61% vs. 34%), less likely to have Medicare (23% vs. 45%; *p* < 0.001), and more likely to experience homelessness (14% vs. 2%; *p* < 0.001). They also had greater overall reliance on government assistance (59% vs. 45%; *p* = 0.002). Individuals with evidence of substance use also had higher rates of smoking (62% vs. 16%; *p* < 0.001) and daily alcohol use (13% vs. 3%; *p* < 0.001). Coronary artery disease did not differ in patients with a history of substance use (32% vs. 30%; *p* = 0.8) (Table 1).

### 3.4. Substance Use and Hospitalization Outcomes

**Figure 1:**
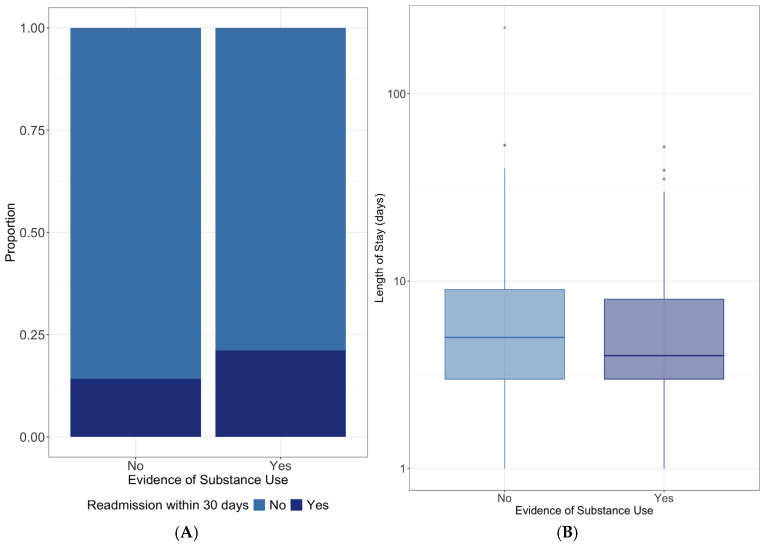
Impact of substance use on 30-day readmission (**A**) and length of stay (**B**) among patients with CHF. A shows the proportion of those readmitted (dark blue) and B shows a boxplot of the length of stay in those who did or did not have evidence of substance use. Differences in rates of 30-day readmission were assessed using a Chi-square test (*p* = 0.048), while differences in length of stay were evaluated using the Wilcoxon rank-sum test (*p* = 0.04).

A one-day decrease in median length of stay was observed among patients with substance use (4 [IQR 3, 8] vs. 5 [IQR 3, 9]; *p* = 0.04; Figure 1). Substance use remained a predictor of length of stay in the multivariable Poisson regression model, after accounting for age, sex, race, CKD, and BMI (IRR = 0.91; (95% CI 0.84, 0.98); *p* = 0.02). Sensitivity analyses including current smoking, a history of COVID-19, or a prior COVID-19 vaccination in the model did not substantially alter the results (*p* < 0.05 for all).

Table 4 shows the prevalence of substance use in patients readmitted after the index admission. As shown in Table 3 and Figure 1, people with evidence of substance use were more likely to be readmitted within 30 days (21% vs. 14%; *p* = 0.048). The association between substance use and 30-day readmission remained significant in the multivariable logistic regression model [aOR 1.8 (95% CI 1.03, 3.0); *p* = 0.039]. Sensitivity analyses accounting for a history of COVID, COVID vaccination, or excluding individuals with a readmission due to COVID did not alter the association of substance use with 30-day readmission (*p* < 0.05 for all).

### 3.5. Follow-Up Appointments and Future Admissions

There were no significant differences between groups in follow-up appointments within 7 days of discharge, suggesting that early outpatient attrition may not explain disparities in readmissions. However, patients with evidence for substance use also had a higher number of hospital readmissions after the index hospitalization (median [IQR]: 2.0 [1.0, 4.0] vs. 1.0 [0.0, 2.0]; *p* < 0.0001). They were also less likely to attend even one follow-up appointment at Cardiology Clinic (41% vs. 51%; *p* = 0.03), despite similar rates of scheduled visits. Substance use remained a predictor of the number of future readmissions in the multivariable Poisson regression model (IRR = 1.7 (95% CI 1.5, 1.9); *p*< 0.0001), accounting for the above covariates plus attendance at any cardiology clinic appointment.

## 4. Discussion

This study examined the prevalence of substance use among individuals with CHF at a single-center safety-net hospital and its association with length of stay, total hospitalizations, and 30-day readmissions, accounting for socioeconomic and demographic factors. These results add to a growing body of evidence that substance use may be an independent risk factor for hospital readmissions in patients with CHF, particularly within underserved populations [14,15,16,17,18,19]. As a safety-net hospital, this setting primarily serves populations that have historically faced structural inequities, economic hardship, and barriers to care, all of which can exacerbate CHF outcomes [16,17,18]. Given that substance use remains a major driver of hospitalizations in the U.S., understanding its impact on CHF readmissions is essential for promoting equitable care [19,20].

Our findings demonstrate that substance use affected 38% of our cohort, which is substantially higher than the ~15.2% prevalence reported by Nishimura et al. in a large registry of over 11,000 patients with heart failure treated at a tertiary academic center in San Diego, and more than double the ~15% prevalence observed by Slim et al. in a retrospective study of predominantly middle-aged adults hospitalized with heart failure at a single tertiary-care center in Texas [14,15]. These differences are most likely due to our broader definition, which included any documented substance use (current or past, by self-report or urine toxicology), compared with prior studies that focused on clinically diagnosed substance use disorders in predominantly insured tertiary-care populations.

Common substances used within our study population included THC, cocaine, methamphetamines, opioids, alcohol, and methadone, with THC and cocaine most frequent. Only five patients self-reported methadone use as treatment for opioid use disorder, while the majority of methadone detections were identified via urine toxicology (Table 2), suggesting most were not using it therapeutically. Individuals with substance use were more likely to be younger, male, African American, experiencing homelessness, enrolled in Medicaid, and current smokers. These patterns align with national data showing disproportionate substance use among structurally marginalized populations [21], though this trend was not observed among Hispanic patients in our cohort. Fourteen percent of individuals with substance use experienced homelessness, consistent with national data on the intersection of housing instability and substance use [22].

A key finding of this study was that substance use is associated with hospital outcomes. Individuals with substance use were more likely to experience 30-day readmissions, higher overall total readmissions, and a slightly shorter median length of stay. These associations remained significant in multivariable models accounting for demographic and clinical covariates. Substance use was also associated with a lower likelihood of attending any scheduled follow-up cardiology appointments, despite similar rates of scheduled visits.

Additionally, socioeconomic status (SES) was strongly linked to substance use among CHF patients. Over half of those with substance use relied on government assistance, nearly all lacked family financial support, and 14% experienced homelessness. Most affected individuals were African American, which may reflect broader structural inequities that increase vulnerability to both substance use and poor health outcomes [22,23]. Our findings extend this literature by demonstrating that these social and economic vulnerabilities intersect to heighten risk for CHF readmissions, suggesting that substance use cannot be considered in isolation from social determinants of health (SDoH). Prior studies have linked neighborhood poverty and limited social support to higher rates of substance use and worse cardiovascular outcomes [24], and our results support these observations in a real-world, safety-net hospital population. These disparities contribute to higher hospital readmission rates, as limited resources can hinder consistent healthcare engagement [14,15,16,17]. National data similarly show elevated substance use among lower-income individuals, highlighting the need for interventions that address both social and economic determinants [25].

Cocaine and opioids emerged as particularly concerning substances, with corresponding 30-day readmission rates of 62% and 32%, respectively. These findings are consistent with prior reports linking stimulant and opioid use to myocardial ischemia, arrhythmias, and worsening heart failure, particularly among African American individuals who already face disproportionate CHF burden and systemic health disparities [25,26,27]. As patients with substance use were less likely to attend follow-up cardiology appointments, it may be beneficial to consider interventions that integrate substance use treatment with CHF management and address barriers to care engagement in-hospital. Embedding routine screening for substance use, linking patients to addiction services, and addressing modifiable SDoH such as housing instability and medication access may reduce readmissions and improve long-term outcomes, particularly in safety-net settings.

### Strengths and Limitations

The strengths of this study lie in its large, unique sample of 500 CHF patients drawn from an underserved, predominantly African American population at a safety-net hospital. By utilizing the CHF Registry, the study ensured standardized data collection, which bolstered the reliability and consistency of the findings. While the study design had significant strengths, there were notable limitations. Urine toxicology screenings were not routinely ordered and were dependent on physician discretion, which may potentially lead to underrepresentation of the true extent of substance use. Nonetheless, the study aimed to examine the impact of any substance use—past or present—on CHF outcomes. Although the time period of the data collection overlapped with the COVID-19 pandemic, inclusion of COVID-related variables in the multivariable models did not alter the association of substance use with CHF outcomes, in part due to the relatively low incidence of COVID-19 prior to the index hospitalization but also likely reflecting the unrelenting nature of chronic diseases.

## 5. Conclusions

By evaluating the prevalence of substance use, patterns of readmission, and interactions with socioeconomic factors, this study highlights the impact of substance use on CHF outcomes, particularly among African American patients facing intersecting challenges related to race, socioeconomic status, and access to care. These findings underscore the need for targeted, equity-focused interventions that address both substance use history and social determinants of health. Potential strategies to reduce readmissions may include harm reduction programs, patient education, linkage to substance use treatment, and interventions targeting social determinants of health. Future research should explore prospective, real-world approaches to evaluate the effectiveness of integrated interventions in improving CHF outcomes and reducing health disparities in underserved communities.

## Figures and Tables

**Table 1:** Baseline Demographics of Patients of the Total Study Sample and Categorized by Substance Use.

Characteristics	Total Study Sample (n = 500)	Evidence of Substance Use n = 189 (38%)	No Substance Use n = 311 (62%)	*p*-Value **
Age, years, mean (SD) *	65 (13)	60 (10)	69 (14)	<0.001
Males, n (%)	279 (56%)	125 (66%)	154 (50%)	0.0003
BMI, kg/m^2^, mean (SD) *	31 (9)	30 (9)	32 (9)	0.04
Insurance Plan, n (%)				<0.001
Medicare	184 (37%)	43 (23%)	141 (45%)	
Medicaid	222 (44%)	116 (61%)	106 (34%)	
Other	61 (12%)	20 (11%)	41 (13%)	
Uninsured	23 (5%)	6 (3%)	17 (5%)	
More than one	9 (2%)	4 (2%)	5 (2%)	
Race, n (%)				0.046
White/Other	14 (3%)	3 (2%)	11 (4%)	
African American	379 (76%)	155 (82%)	224 (72%)	
Hispanic	58 (12%)	14 (7%)	44 (14%)	
Unknown	49 (10%)	17 (9%)	32 (10%)	
Living Condition, n (%)				<0.001
Permanent Residence	434 (87%)	151 (80%)	283 (91%)	
Homeless	33 (7%)	27 (14%)	6 (2%)	
Extended Care Facility	15 (3%)	5 (3%)	10 (3%)	
Unable to Determine	18 (4%)	6 (3%)	12 (4%)	
Income, n (%)				0.002
Employed	55 (11%)	15 (8%)	40 (13%)	
Pension	12 (2%)	0 (0%)	12 (4%)	
Government Assistance	253 (51%)	112 (59%)	141 (45%)	
Family Support	35 (7%)	10 (5%)	25 (8%)	
None	115 (23%)	42 (22%)	73 (23%)	
Unable to Determine	30 (6%)	10 (5%)	20 (6%)	
Comorbidities, n (%)				
Hypertension, n (%)	452 (90%)	172 (91%)	280 (90%)	0.8
Diabetes mellitus, n (%)	231 (46%)	74 (39%)	157 (50%)	0.02
Dyslipidemia, n (%)	231 (46%)	84 (44%)	147 (47%)	0.6
Coronary Artery Disease, n (%)	154 (31%)	60 (32%)	94 (30%)	0.8
History of Atrial Fib/Flutter, n (%)	112 (22%)	31 (16%)	81 (26%)	0.01
Sleep Apnea, n (%)	71 (14%)	22 (12%)	49 (16%)	0.2
COPD/asthma, n (%)	179 (36%)	85 (45%)	94 (30%)	0.001
History of Renal Disease, n (%)	214 (43%)	73 (39%)	141 (45%)	0.2
Obesity, n (%)	238 (48%)	78 (41%)	160 (51%)	0.03
Anemia, n (%)	262 (52%)	98 (52%)	164 (53%)	0.8
CVA or TIA, n (%)	95 (19%)	30 (16%)	65 (21%)	0.2
Peripheral Vascular Disease, n (%)	55 (11%)	17 (9%)	38 (12%)	0.3
Valvular Heart Disease, n (%)	75 (15%)	17 (9%)	58 (19%)	0.003
CHF diagnosis prior to IH, n (%)	321 (64%)	127 (67%)	194 (62%)	0.3
Current cigarette smoker, n (%)	166 (33%)	117 (62%)	49 (16%)	<0.001
Daily alcohol use, n (%)	33 (7%)	24 (13%)	9 (3%)	<0.001

Statistics presented: mean (SD) *; n (%). ** Statistical tests performed: Wilcoxon rank-sum test for continuous variables; Fisher’s exact test for categorical variables. Abbreviations: Body Mass Index (BMI), Cerebrovascular Accident (CVA), Chronic Obstructive Pulmonary Disease (COPD), Transient Ischemic Attack (TIA), Index Hospitalization (IH).

**Table 2:** Types of Substances Used by CHF Patients with Evidence for Substance Use.

Substance Used	Overall Substance Use Prevalence, n = 189	All-Time Prevalence by UTOX n = 134	All-Time Prevalence by Social History n = 124
Barbiturate, n (%)	3 (2)	3 (2)	0 (0)
Benzodiazepine, n (%)	26 (14)	26 (19)	2 (2)
Cocaine, n (%)	118 (62)	79 (59)	108 (87)
Methadone, n (%)	24 (13)	22 (16)	5 (4)
Opiates, n (%)	59 (31)	43 (32)	40 (32)
PCP, n (%)	4 (2)	3 (2)	1 (1)
THC, n (%)	119 (63)	37 (28)	113 (91)
Amphetamines, n (%)	6 (3)	6 (4)	0 (0)
Other, n (%)	1 (0.5)	0 (0)	1 (1)

Substance use in CHF admissions (n = 500) was identified in 189 patients (134 individuals had positive urine toxicology and 124 had a positive social history); 311 had no evidence of substance use. Note that some individuals had both a positive urine toxicology and a positive social history and that some individuals used more than one substance. Abbreviations: CHF, congestive heart failure; Utox, urine toxicology; PCP, phencyclidine; THC, tetrahydrocannabinol.

**Table 3:** Admissions, Readmissions, and Appointments Stratified by Evidence of Substance Usage.

	Total Patients (n = 500)	No Substance Use n = 311 (62%) *	Evidence of Substance Use n = 189 (38%) *	*p*-Value **
LOS of index hospitalization	5.0 [3.0, 8.0]	5.0 [3.0, 9.0]	4.0 [3.0, 8.0]	0.04
Attendance at any follow-up appointment at Harlem Cardiology Clinic	238 (48%)	160 (51%)	78 (41%)	0.03
Readmitted within 30 days of index hospitalization	84 (17%)	44 (14%)	40 (21%)	0.048
30 day readmission related to CHF	61 (12%)	32 (10%)	29 (15%)	0.12
Total number of hospital readmissions after index hospitalization	1.0 [0.0, 3.0]	1.0 [0.0, 2.0]	2.0 [1.0, 4.0]	<0.0001

* Statistics presented: n (%); median [IQR]. ** Statistical tests performed: Wilcoxon rank-sum test; Fisher’s exact test.

**Table 4:** Type of Substance Usage and Hospital Readmission Rate Among CHF Patients.

Substance Used	Readmission Rate at 30 Day, n (%)	All Time Readmission Rate, Median [IQR]
Barbiturates, n = 3, n (%)	1 (33%)	10.0 [0.0, 16.0]
Benzodiazepines, n = 26, n (%)	9 (35%)	3.0 [0.0, 6.0]
Cocaine, n = 118, n (%)	29 (25%)	2.0 [0.5, 4.0]
Methadone, n = 24, n (%)	10 (42%)	3.5 [1.0, 5.5]
Opiates, n = 59, n (%)	19 (32%)	2.0 [1.0, 5.0]
PCP, n = 4, n (%)	0 (0%)	2.0 [0.5, 3.5]
THC, n = 119, n (%)	18 (15%)	1.0 [0.0, 4.0]
Amphetamines, n = 6, n (%)	0 (0%)	3.0 [0.0, 8.0]
Daily Alcohol, n = 33, n (%)	5 (15%)	1.0 [0.0, 3.0]
Current Smoker, n = 166, n (%)	33 (20%)	2.0 [0.0, 4.0]
Any Substance Use, n = 189, n (%)	40 (21%)	2.0 [1.0, 4.0]
No Overall Substance Use, n = 311, n (%)	44 (14%)	1.0 [0.0, 2.0]

Abbreviations: PCP, phencyclidine; THC, tetrahydrocannabinol.

## Data Availability

Data cannot be shared due to IRB stipulations and concerns regarding the privacy and confidentiality of study subjects.
